# Effect of High Temperature on Abamectin and Thiamethoxam Tolerance in *Bemisia tabaci* MEAM1 (Hemiptera: Aleyrodidae)

**DOI:** 10.3390/insects15060399

**Published:** 2024-05-29

**Authors:** Mi Zhou, Yuncai Liu, Yucheng Wang, Yawen Chang, Qingjun Wu, Weirong Gong, Yuzhou Du

**Affiliations:** 1 Institute of Applied Entomology, College of Plant Protection, Yangzhou University, Yangzhou 225009, China; zhoumi20011101@126.com (M.Z.); liuyuncai990@163.com (Y.L.); wangycyzu@163.com (Y.W.); changyawen@yzu.edu.cn (Y.C.); 2 Institute of Vegetables and Flowers, Chinese Academy of Agricultural Sciences, Beijing 100081, China; wuqingjun@caas.cn; 3 Plant Protection and Quarantine Station of Jiangsu Province, Nanjing 210036, China; 15861351327@163.com; 4 Joint International Research Laboratory of Agriculture and Agri-Product Safety, The Ministry of Education, Yangzhou University, Yangzhou 225009, China

**Keywords:** *Bemisia tabaci*, CYP450s, insecticide tolerance, high temperature, thiamethoxam, abamectin

## Abstract

**Simple Summary:**

*Bemisia tabaci* (Gennadius) is a worldwide agricultural pest and is one of the most harmful invasive insect pests in the world. The overuse of insecticides has caused *B. tabaci* to develop high resistance against multiple insecticidal chemicals. It is well recognized that over-expression of cytochrome P450s involved in insecticide detoxification is one common and major mechanism of resistance to various classes of insecticides in insects. In addition to gene duplication and amplification, up-regulation of P450s at the transcriptional level is a common and important mechanism for P450-mediated insecticide resistance. Here, two new CYP450 genes from *B. tabaci* MEAM1 were cloned, and their expression patterns were studied in response to two different insecticides, high temperature, and their combined effects. The results showed that the insecticide tolerance could be reduced by high temperatures; under combined stress, the mortality of *B. tabaci* MEAM1 increased. This study is an important addition to the research on insecticide tolerance in *B. tabaci* and may help to reduce the overreliance on insecticides for *B. tabaci* control.

**Abstract:**

*Bemisia tabaci* (Gennadius) is one of the most important invasive species in China, with strong insecticide resistance and thermotolerance. In this study, we investigated the effects of elevated temperature on the tolerance of *B. tabaci* MEMA1 to abamectin (AB) and thianethixam (TH) insecticides. We firstly cloned two new CYP450 genes from *B. tabaci* MEAM1, including one CYP6 family gene (*BtCYP6k1*) and one CYP305 family gene (*BtCYP305a1*). The expression patterns of the two BtCYP450 genes were compared in response to high-temperature stress and insecticide exposure, and RNAi was then used to demonstrate the role that these two genes play in insecticide tolerance. The results showed that expression of the two BtCYP450 genes could be induced by exposure to elevated temperature or insecticide, but this gene expression could be inhibited to a certain extent when insects were exposed to the combined effects of high temperature and insecticide treatment. For AB treatment, the expression of the two BtCYP450 genes reached the lowest level when insects were exposed to a temperature of 41 °C and treated with AB (combined effects of temperature and insecticide). In contrast, TH treatment showed a general decrease in the expression of the two BtCYP450 genes with exposure to elevated temperatures. These findings suggest that insecticide tolerance in *B. tabaci* MEAM1 could be mediated by high temperatures. This study provides a prospective method for the more effective application of insecticides for the control of *B. tabaci* in the field.

## 1. Introduction

*Bemisia tabaci* (Gennadius) is a widespread agricultural pest, distributed all over the world except for Antarctica. It is one of the most harmful invasive pests in the world, wreaking havoc on Solanacea, Cruciferaceae, Cucurbitaceae, and other crops [1]. There are three main ways through which *B. tabaci* can damage crops: (a) the direct ingestion of plant sap, which causes the plant to grow slowly and wilt in severe cases; (b) induction of the secretion of honeydew, which induces sooty mold infections and reduces the plant’s photosynthetic efficiency; and (c) the transmission of plant viruses, especially begomoviruses and criniviruses, which cause plant dwarfing, reduced seed setting, and even plant death [2]. *B. tabaci* is a cryptoactive complex containing at least 44 cryptic species [3]. Within this species complex, two cryptic species, Middle East–Asia Minor 1 (MEAM1) and Mediterranean (MED), formerly often referred to as the B and Q “biotypes”, are the two most influential invasive *B. tabaci* cryptospecies [4].

At present, the prevention and control of *B. tabaci* is still mainly based on chemical control, especially environmentally friendly insecticides such as neonicotinoids. The mechanisms of insect resistance to insecticides mainly include epidermal penetration resistance, target resistance, and detoxification. The main reason for the emergence of pesticide resistance is the improvement of insects’ ability to detoxify and metabolize insecticides [5]. The main enzyme involved in metabolism and detoxification processes is cytochrome P450 monooxygenase (*CYP450*) [6,7]. Cytochrome P450 monooxygenase is a class of heme and thiolhydroxyl protein widely present in living organisms. P450 is present in all life forms, and it has the two functions of catalyzing the metabolism of many exogenous substances (such as drugs entering the body and toxic substances in the environment) and transforming endogenous substances (such as fatty acids, cholesterol, and steroid hormones) into essential active substances for living organisms [8]. Studies have shown that the resistance of Q-type *B. tabaci* to nicotinoid insecticides is related to microsomal cytochrome P450 multifunctional oxidase [9]. Karunker et al. (2008) also found that resistance to imidacloprid insecticides in *B. tabaci* B and Q was positively correlated with the expression level of the P450 CYP6CM1 gene in vivo [10]. Multiple studies have shown that the metabolic activity of the P450 enzyme system is the main mechanism by which most insects develop insecticide resistance [11,12].

Under natural conditions, temperature is one of the most important environmental factors affecting the growth and development of insects [13]. It is also an important factor affecting the resistance of insects to insecticides. High-temperature stress leads directly to protein denaturation and inactivation and water loss in insects, while low-temperature stress causes the cells to freeze and rupture, thereby causing irreversible damage and even death to insects [14]. Previous studies have found that high temperatures affect insects’ resistance to insecticides, and this phenomenon has been reported for many insects, including *Plutella xylostella*, *Tetranychus cinnabarinus*, and *Spodoptera littoralis* [15,16,17]. In *Spodoptera littoralis*, the susceptibility to lambda-cyhalothrin and methomyl increased with increasing temperature [17]. Also, increased temperature could regulate the tolerance of *B. tabaci* MED to the insecticide thiamethoxam and was associated with the induction of *CYP6CM1* [18]. However, in brown planthoppers and *Drosophila*, there was no strong correlation between the activities of the detoxifying enzymes and temperature, while the quantity of reactive oxygen species (ROS) increased with temperature [19]. Previous research has shown that temperature tolerance varies significantly between the two invasive whiteflies and the MED species is more tolerant to high temperatures than the MEAM1 species, especially in the adult stage [20]. Therefore, studying the effect of high temperature on the resistance of *B. tabaci* is of great guiding significance for understanding the invasion mechanism and inferring related predictions.

In this study, we explored the effect of high temperatures on insecticide tolerance in *B. tabaci*. We first identified two new CYP450 genes from *B. tabaci* MEAM. Then, the expression patterns of these two BtCYP450 genes were compared under different high temperatures and insecticide treatments to study the interactions between the BtCYP450 genes and temperature stress. Also, the role of CYP450 in regulating insecticide tolerance in *B. tabaci* MEAM1 was explored via RNAi.

## 2. Materials and Methods 

### 2.1. Insects

The *B. tabaci* MEAM1 line used in this research were continuously reared in our laboratory for multiple generations. The MEAM1 cryptic species of *B. tabaci* was identified via the specific primers described by Liu (2017) [21]. Then, colonies were established indoors on tomato (*Lycopersicon esculentum*) plants for multiple generations without exposure to any insecticides. The growth chamber was set at 26 ± 1 °C, with a 16 L: 8D photoperiod and 60–70% relative humidity.

### 2.2. RNA Isolation and Cloning Experiments

Total RNA was extracted from *B. tabaci* MEAM1 adults using the RNA-easy^TM^ Reagent (Vazyme Biotech, Nanjing, China), and the samples were stored at −80 °C until needed. Full-length cDNAs of genes encoding CYP450s were obtained by means of 5′- and 3′-RACE (SMART RACE, Clontech, Mountain View, CA, USA) using the primers shown in Table S1. 

Two putative CYP450 genes were identified based on the transcriptome data [22]; the gene-specific primers used to amplify these genes are listed in Table S1. PCR products were separated using a gel extraction kit (Axygen, Union City, CA, USA) and then cloned into pGEM-T Easy vector (Promega, Fitchburg, WI, USA). Ligation reaction was then used to transfer these genes into competent *Escherichia coli* DH5α cells for sequencing. Identification of the open reading frames (ORFs) was performed using the online software ORF Finder “https://www.ncbi.nlm.nih.gov/orffinder/ (Accessed: 17 September 2023)”. The physical and chemical properties of the two genes, such as the amino acid sequence translation, relative molecular weight, isoelectric point, etc., were identified using the online software Translate tool “http://web.expasy.org/translate/ (Accessed: 17 September 2023)”. The homology was searched with Blast in NCBI, and MEGA7.0 was used to construct the phylogenetic tree of the two CYP450 family genes of *B. tabaci* MEAM1 and the CYP450 family genes of *B. tabaci* and other species, based on the adjacency method.

### 2.3. Insecticide Bioassays

The pesticide used for bioassays included 1.8% *w*/*w* abamectin (AB, Jinan Zhongkekeji Green Bioengineering Co., Ltd., Jinan, China) and 30% *w*/*w* thiamethoxam (TH, Jiangsu Jianpai Chemical Industry Co., Ltd., Suzhou, China). The lethal concentrations (LC_50_) of abamectin and thiamethoxam for *B. tabaci* MEAM1 were determined via a feeding bioassay: formulated insecticides were diluted with 30% sucrose solution to different concentrations (for AB, it was 0–2 mg·L^−1^, and for TH, it was 0–15 mg·L^−1^) with a targeted mortality ranging from 0% to 100%. Fifty *B. tabaci* MEAM1 adults were randomly placed and transferred into a special feeding tube, one side of which was filled with 300 μL of sucrose solution containing different concentrations of pesticide for feeding while the other side was covered with a screen for ventilation. Adults placed in feeding tubes and fed only with 30% sucrose solution were used as controls. The tubes containing the experimental insects were placed in an artificial climate chamber and maintained at the same relative temperature, humidity, and photoperiod as the colony. The mortality rate was recorded after 24 h. Each treatment consisted of three biological replicates.

### 2.4. Temperature Treatments

Fifty *B. tabaci* MEAM1 adults were placed in centrifuge tubes and exposed to temperatures of 37 °C, 39 °C, and 41 °C, respectively, in a thermostatic bath (DC-3010; Jiangnan Equipment, Ningbo, China) for 1 h, and the insects were then left to recover at 26 °C for 1 h after treatment. Control insects were treated at 26 °C, with each treatment including three biological replicates.

### 2.5. Combined Temperature and Insecticide Treatment

When treated with both elevated temperature and insecticide exposure, the *B. tabaci* MEAM1 adults were firstly exposed to high temperatures (37 °C, 39 °C, or 41 °C) for 1 h, then survivors were transferred to the feeding tube with the LC_50_ dose of the insecticide and placed in an artificial climate chamber. The other treatment groups were exposed to the LC_50_ dose of insecticide and then treated with different high temperatures for 1 h. Mortality was recorded after combined treatment. The two different treatments used the same control: adults exposed to the LC_50_ dose of the insecticide. Each treatment included three biological replicates.

### 2.6. RNA Interference

RNA interference analysis was conducted to examine whether the significantly upregulated expression of the two CYP450 genes in *B. tabaci* MEAM1 played a role in the resistance to abamectin and thiamethoxam. Synthesis of the two *CYP450s’* dsRNA and *green fluorescent protein* (GFP) dsRNA in vitro was based on the instructions of the Thermo Scientific^TM^ TranscriptAid^TM^ T7 High Yield Transcription Kit (Thermo, Waltham, MA, USA). All primers designed to generate dsRNA are listed in Table S1. dsRNA was stored at −80 °C before use.

RNA interference was conducted through directly feeding dsRNA to *B. tabaci* MEAM1 adults in the feeding tube for 24 h, as described in Section 2.3. dsCYP450 and dsGFP (100 ng/μL) were diluted in 30% (*w*/*v*) sucrose for use in the experiments. The effiency of RNA interference was tested 24 h post-feeding. Then, the survivors were transferred to the feeding tube with the LC_50_ dose of insecticide for 24 h. The mortality rate was recorded 24 h post-feeding. *B. tabaci* MEAM1 adults treated with dsGFP and the LC_50_ dose of the insecticide were used as the control group. Each treatment included three biological replicates.

### 2.7. Quantitative Real-Time PCR

The two *CYP450s* (*BtCYP6k1* and *BtCYP305a1*) were analyzed via real-time quantitative PCR (qPCR), and template cDNA synthesis was performed using Hiscript^®^ III (Vazyme, Nanjing, China). The RT SuperMix for qPCR (+gDNA wiper) real-time quantitative reverse transcription kit was employed, and qPCR amplification reaction was performed using the SYBR Green I (Vazyme, Nanjing, China) chimeric fluorescence method. We selected two genes stably expressed in different types of cells or tissues of *B. tabaci* MEAM1 as reference genes, and the 2^−ΔΔCt^ method was used to calculate expression [23]. *RPL29* (60S ribosomal protein L29) and *EF-1a* (Elongationfactor 1 alpha) were selected as internal reference genes [24].

### 2.8. Data Analysis

LC_50_ values were evaluated using DPS v. 9.01 [25]. Significant differences in mortality and gene expression were determined using one-way ANOVA, followed by Tukey’s multiple comparison and analysis with SPSS v. 16.0. Student’s *t*-test was used for the two-mean comparisons of mortalities and gene expression after feeding on dsRNA. Results were considered significant at *p* < 0.05. The data were represented as means ± standard errors.

## 3. Results

### 3.1. Sequence and Phylogenetic Analysis of P450s in B. tabaci MEAM1

Two new CYP450 genes were cloned from *B. tabaci* MEAM1, named *BtCYP6k1* and *BtCYP305a1*, and deposited in GenBank under the accession numbers PP239342 and PP239341, respectively (Table S2). Comparing the deduced proteins using the GenBank and PROSITE databases, it was found that they had high similarity to CYP450 family genes and contained signature sequences that were identified in the deduced amino acid sequences of *BtCYP450s*. 

**Figure 1:**
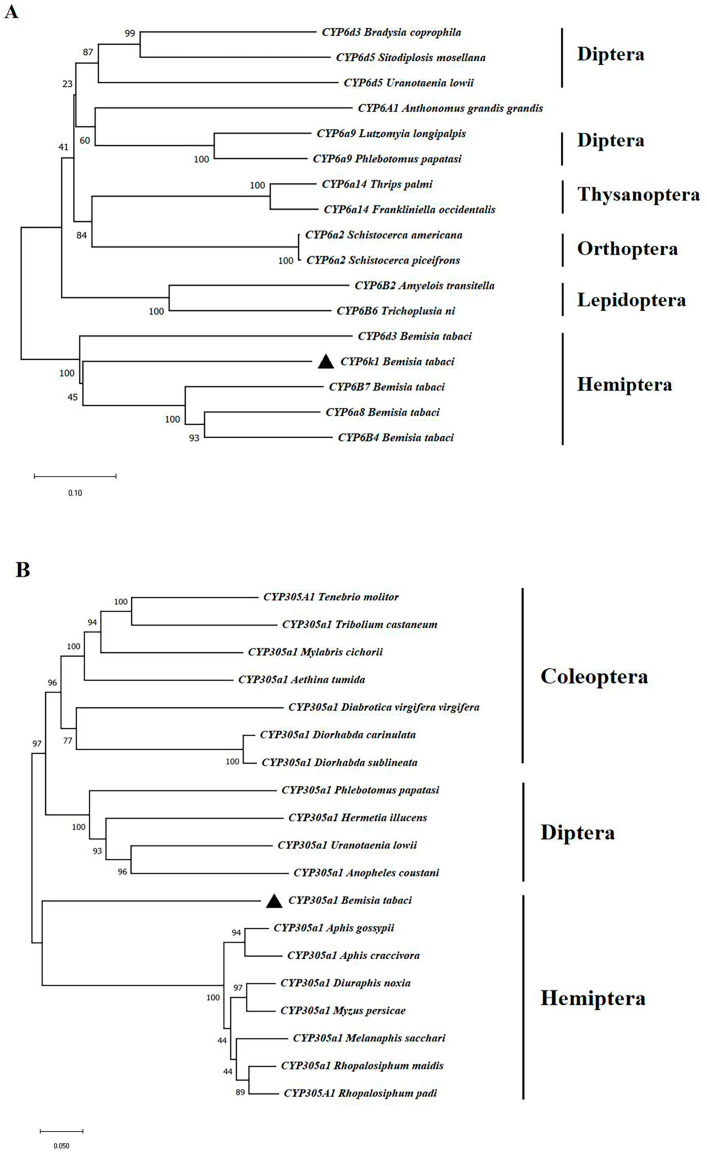
Phylogenetic trees of two CYP450 amino acid sequences. Panels indicate the phylogenetic relationship of A (*CYP6k1*) and B (*CYP305a1*). The tree was obtained using the neighbor-joining method and predicted amino acid sequences of CYP450s in orders Hemiptera, Coleoptera, Lepidoptera, Diptera, Thysanoptera, and Orthoptera.

The phylogenetic tree exhibiting the phylogenetic relationships between the two *BtCYP450s* and *CYP450s* from other insects was constructed via the neighbor-joining method using orthologues. The results indicate that the two new *BtCYP450s* were highly similar to those in other Hemipteran insects (Figure 1).

### 3.2. Toxicity Assessments of Two Insecticides

Based on mortality data with different insecticide concentrations, a regression analysis was carried out to establish the dose–response curves. The LC_50_ values of AB and TH for *B. tabaci* adults were 0.151 and 2.134 mg/L, respectively (Table 1). This indicated that the insecticide sensitivity of *B. tabaci* MEAM1 to AB was stronger than that to TH.

### 3.3. Mortality of B. tabaci MEAM1 in Response to High Temperature and Insecticide Exposure

**Figure 2:**
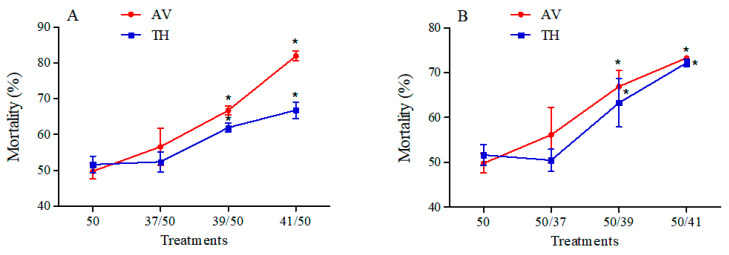
Mortality of *B. tabaci* MEAM1 subjected to high temperature and insecticides. Panels indicate pre-treatment with high temperature then LC_50_ insecticide exposure (**A**), and pre-treatment with LC_50_ insecticide exposure then high temperature (**B**). Asterisks indicate significant differences in mortality during combined stress in *B. tabaci* compared with the control at 26 °C. Abbreviations: 50 indicates LC_50_ insecticide exposure; 37/50, 39/50, and 41/50 represent 37/39/41 °C and LC_50_ insecticide exposure; 50/37, 50/39, and 50/41 represent LC_50_ insecticide exposure and 37/39/41 °C.

In general, mortality rates increased when *B*. *tabaci* MEAM1 specimens were treated with high temperature and insecticide (Figure 2). When insects were pre-treated with high temperature, the mortality increased as the temperature increased and reached its highest level at 41 °C, while it increased by 32.17% for AV and 15.16% for TH (Figure 2A; AV: *F*_3,8_ = 22.123, *p* < 0.05; TH: *F*_3,8_ = 11.058, *p* < 0.05). The mortality rates showed similar trends when insects were pre-treated with high temperature and when they were pre-treated with insecticides, and the highest mortality (73.32% for AV and 72.23% for TH) was observed at 41 °C (Figure 2B; AV: *F*_3,8_ = 8.1, *p* < 0.05; TH: *F*_3,8_ = 11.649, *p* < 0.05).

### 3.4. Expression of BtCYP450s in Response to Thermal Stress

**Figure 3:**
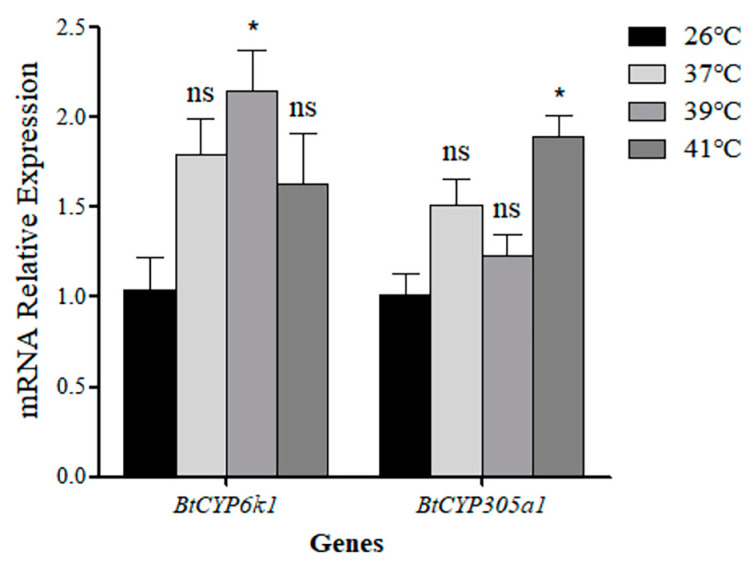
Expression of two *CYP450s* during high-temperature stress. Asterisks indicate significant differences between temperature treatments and control (26 °C). Abbreviation: ns, no significant difference. Tukey’s multiple range test was used for pairwise comparison of means (*p* < 0.05).

qRT-PCR was used to detect the expression of two *BtCYP450s* during high temperature exposure. The results showed that the expression levels of *BtCYP6k1* and *BtCYP305a1* were significantly up-regulated in response to the high temperatures of 39 °C and 41 °C, respectively, being 2.15-fold and 1.87-fold higher than the control (26°C). Meanwhile, the expression level of *BtCYP6k1* at 37 °C and 41 °C and the *BtCYP305a1* expression level at 37 °C and 39 °C showed no significant differences when compared with the control (Figure 3; *BtCYP6k1: F*_3,8_ = 4.164, *p* < 0.05; *BtCYP305a1: F*_3,8_ = 9.216, *p* < 0.05). 

### 3.5. Expression of BtCYP450s in Response to Insecticide Stress

**Figure 4:**
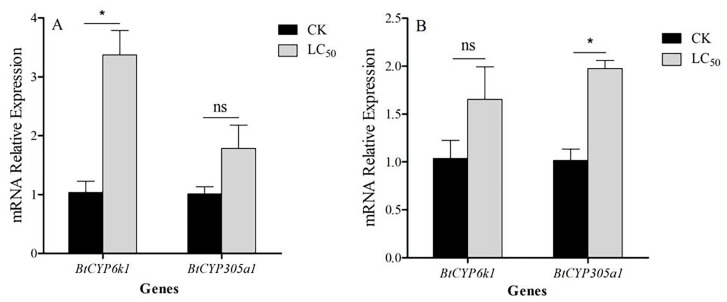
Expression of two CYP450 genes in *B. tabaci* exposed to abamectin and thiamethoxam. Panels indicate exposure to (**A**) abamectin and (**B**) thiamethoxam. CK means blank control and LC_50_ means LC_50_ insecticide exposure. Asterisks indicate significant differences in *CYP450* expression in *B. tabaci* during insecticide stress compared with the control.

The expression patterns of the two BtCYP450 genes under insecticide treatment differed between AB and TH exposures. Compared with the control, the expression of *BtCYP6k1* was significantly up-regulated by AB, being 3.37-fold higher than the control, whereas *BtCYP305a1* showed weak expression (Figure 4A; *BtCYP6k1: t* = 3.182, *p* < 0.05; *BtCYP305a1: t* = 1.87, *p* = 0.135). For TH stress, the expression of *BtCYP305a1* was significantly up-regulated 1.98-fold, but *BtCYP6k1* remained non-responsive (Figure 4B; *BtCYP305a1: t* = 6.517, *p* < 0.05; *BtCYP6k1: t* = 1.574, *p* = 0.191).

### 3.6. Expression of BtCYP450s in Response to Combined Stress of High Temperature and Insecticide 

**Figure 5:**
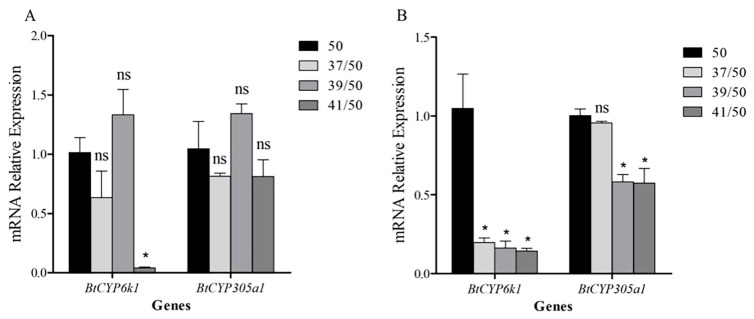
Relative expression levels of two *CYP450s* in *B. tabaci* subjected to high temperatures followed by insecticide treatment. Panels indicate exposure to (**A**) abamectin and (**B**) thiamethoxam. Asterisks indicate significant differences in *CYP450* expression in *B. tabaci* subjected to heat stress compared with the control at 26 °C. Abbreviations: ns indicates no significant difference; 50 indicates LC_50_ insecticide exposure; 37/50, 39/50, and 41/50 indicate 37/39/41 °C and LC_50_ insecticide exposure.

**Figure 6:**
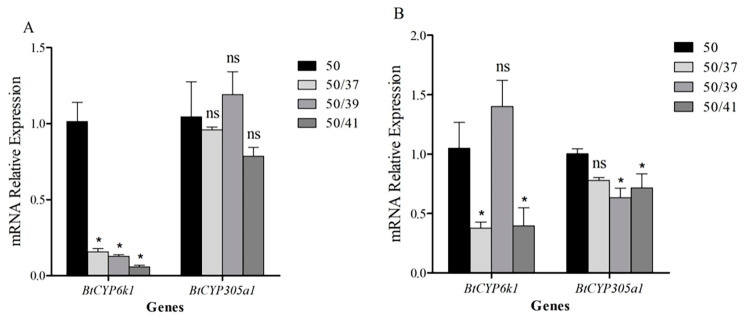
Relative expression levels of two *CYP450s* under heat stress after insecticide pretreatment. Panels indicate exposure to (**A**) abamectin and (**B**) thiamethoxam. Asterisks indicate significant differences in the expression of two *CYP450s* under insecticide pretreatment. Abbreviations: ns indicates no significant difference; 50 indicates LC_50_ insecticide exposure; 50/37, 50/39, and 50/41 indicate LC_50_ insecticide exposure and 37/39/41 °C.

When compared with only AB treatment, the expression of *BtCYP6k1* in response to AB decreased significantly after pre-treatment at 41 °C, dropping to 0.04-fold (Figure 5A; *BtCYP6k1: F*_3,8_ = 11.054, *p* < 0.05), while pre-treatment with AB significantly induced the expression of *BtCYP6k1* in response to the three elevated temperatures, which dropped to 0.16-, 0.13-, and 0.06-fold, respectively (Figure 6A; *BtCYP6k1: F*_3,8_ = 49.597, *p* < 0.05). However, the expression of *BtCYP305a1* in response to the combined stress of high temperature and AB was not significantly different from the control (*BtCYP305a1* in Figure 5A: *F*_3,8_ = 3.144, *p* = 0.087; *BtCYP305a1* in Figure 6A: *F*_3,8_ = 1.448, *p* = 0.3). 

For thiamethoxam, pre-treatment with the three high temperatures resulted in a significant reduction in the expression of *BtCYP6k1* in response to TH; compared with the control, they dropped to 0.19-, 0.16-, and 0.14-fold, respectively (Figure 5B; *BtCYP6k1: F*_3,8_ = 15.491, *p* < 0.05), whereas the expression level of *BtCYP6k1* was significantly lower than the control only at 37 and 41 °C after pre-exposure to insecticide, dropping to 0.38-fold at 37 °C and 0.4-fold at 41 °C (Figure 6B; *BtCYP6k1: F*_3,8_ = 11.149, *p* < 0.05). Compared with only TH treatment, the expression of *BtCYP305a1* was significantly down-regulated when pre-treated at 39 and 41 °C, with reductions of 0.58- and 0.57-fold, respectively (Figure 5B; *BtCYP305a1: F*_3,8_ = 16.836, *p* < 0.05), and the expression pattern of *BtCYP305a1* in response to exposure to heat stress followed by TH was similar (Figure 6B; *BtCYP305a1:* 50/39: 0.63-fold, 50/41: 0.72-fold, *F*_3,8_ = 4.375, *p* < 0.05).

### 3.7. Effect of RNAi on Tolerance to Insecticides

**Figure 7:**
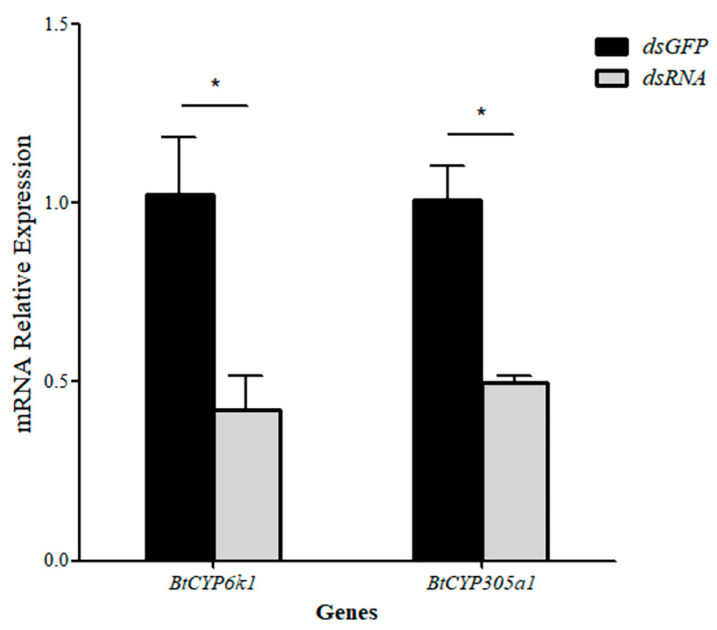
Efficiency of RNA knockdown of two *BtCYP450s*. Asterisks denote significant differences between the RNAi treatment and controls.

After 24 h of feeding on the sucrose solution containing dsRNA specific for the two *BtCYP450s* (*BtCYP6k1* and *BtCYP305a1*), the expression levels decreased by 58% and 50%, respectively (Figure 7: *BtCYP6k1: t* = 3.222, *p* < 0.05; *BtCYP305a1: t* = 5.305, *p* < 0.05).

**Figure 8:**
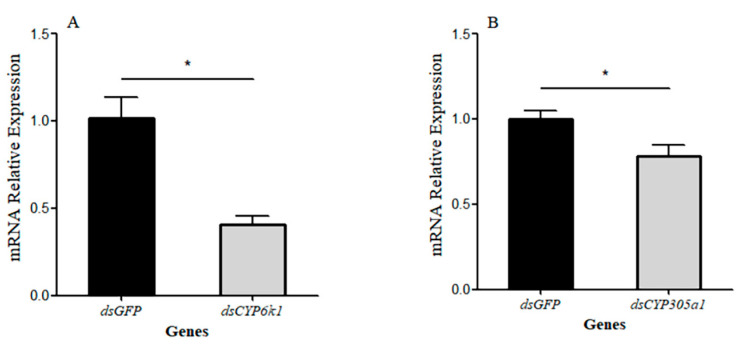
Functional analysis of two *BtCYP450s* via RNAi. (**A**) Expression of *BtCYP6k1* in response to abamectin stress after feeding on the corresponding dsRNA for 24 h; (**B**) expression of *BtCYP305a1* in response to thiamethoxam stress after feeding on the corresponding dsRNA for 24 h. Asterisks denote significant differences between the RNAi treatment and controls.

**Figure 9:**
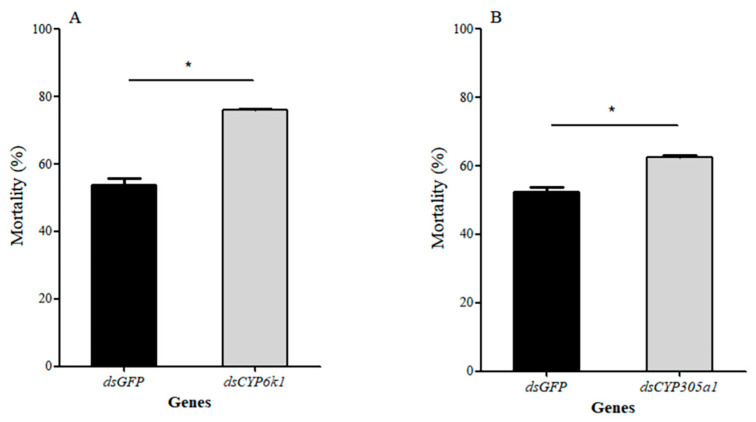
Functional analysis of two *BtCYP450s* via RNAi. (**A**) Mortality of *B. tabaci* MEAM1 in response to abamectin stress after 24 h of feeding on the corresponding dsRNA; (**B**) mortality of *B. tabaci* MEAM1 in response to thiamethoxam stress after 24 h of feeding on the corresponding dsRNA. Asterisks denote significant differences between the RNAi treatment and controls.

The function of the two *BtCYP450s* was investigated by supplying insects with dsCYP6k1 and dsCYP305a1 and exposing *B. tabaci* MEAM1 to insecticides (Figure 8 and Figure 9). Twenty-four hours after feeding with dsCYP6k1 and dsCYP305a1, the expression levels of *BtCYP6k1* in response to abamectin at LC_50_ were reduced by approximately 59%, while the expressions levels of *BtCYP305a1* in response to thiamethoxam at LC_50_ were reduced by approximately 22%, with respect to the dsGFP control (Figure 8A: *BtCYP6k1*: *t* = 4.611, *p* < 0.05; Figure 8B: *BtCYP305a1*: *t* = 3.413, *p* < 0.05). Furthermore, the mortality of *B. tabaci* MEAM1 fed with dsCYP6k1 or dsCYP305a1 was significantly increased, with increases of 22% and 10%, respectively (Figure 9A: *BtCYP6k1: t* = 11.525, *p* < 0.05; Figure 9B: *BtCYP305a1*: *t* = 6.395, *p* < 0.05).

## 4. Discussion

CYP450 genes are involved in the metabolism of endogenous substances and exogenous substances including natural and synthetic toxic compounds [26,27]. In insects, many *P450s* are able to metabolize a variety of exogenous substances that lead to resistance [28]. Research has demonstrated that the over-expression of CYP450 genes increased the metabolism of pesticides, contributing to insecticide resistance or tolerance in invertebrates [29].

Based on our previous transcriptome data, we identified two new CYP450 genes in *B. tabaci* MEAM1 and analyzed their sequence characteristics [22]. According to the naming conventions for CYP450 genes, they were named *BtCYP6k1* and *BtCYP305a1* [30,31]. The predicted amino acid sequences of *BtCYP450s* showed high similarity to conserved sequences in *CYP450s* from other hemipteran insects. Phylogenetic analysis indicated that the two new *BtCYP450s* could be distinguished from the other orthologous proteins in other insects (Figure 1), including Hemiptera, Coleoptera, Lepidoptera, Diptera, Thysanoptera, and Orthoptera, and grouped with *CYP450s* in other hemipteran insects including *Aphis gossypii*, *Aphis craccivora, Diuraphis noxia*, *Mvzus persicae*, *Melanaphis sacchari*, *Rhopalosiphum maidis*, and *Rhopalosiphum padi*.

There is strong evidence that insecticide resistance is often conferred by cytochrome p450 monooxygenases [32]. For instance, CYP4 and CYP6 families are associated with imidacloprid tolerance in *B. tabaci* MED [33], while the *CYP6DB3* gene was also over-expressed in thiamethoxam-resistant *B. tabaci* populations and the expression of *CYP6DB3* can be induced under thiamethoxam selection pressure [34]. Furthermore, the P450 monooxygenase was found to be conducive to the development of abamectin resistance [35]. In our study, the expression of *BtCYP6k1* was significantly influenced when exposed to abamectin and *BtCYP305a1* by exposure to thiamethoxam (Figure 4), and this was verified with RNAi experiments (Figure 8 and Figure 9). These results indicate that *BtCYP6k1* and *BtCYP305a1* are involved in the tolerance of *B. tabaci* to abamectin and thiamethoxam. 

High temperature has been shown to have different effects in the adaptation of various insects to stress [23,36,37]. In our study, thermal stress was found to induce the expression of *BtCYP450s* but had a significant effect on the expression of *BtCYP6k1* only at 39 °C and *BtCYP305a1* only at 41 °C (Figure 3). However, the expression levels of two *BtCYP450s* declined after pre-treatment with elevated temperatures. Similar results have been observed in *Plutella xylostella* (L.) (Lepidoptera: Plutellidae), which showed high resistance during spring and autumn and low resistance during summer [15]. Prior reports indicate that *CYP6CM1*, *CYP6CX1*, and *CYP4C64* expression levels in *B. tabaci* MED increased when specimens were exposed to a temperature of 31 °C and decreased when they were exposed to temperatures of 35 °C or higher [18]. 

In our previous study, we found that the transcription levels of several *BtCYP450s* in *B. tabaci* MED were elevated during thermal stress, potentially leading to increased tolerance to insecticides [38]. Similarly, *Liriomyza trifolii* exhibits adaptive cross-tolerance to high temperature and abamectin [39]. However, the *BtCYP450s* in *B. tabaci* MEAM1 showed different results (Figure 5 and Figure 6). Furthermore, mortality rates in *B. tabaci* increased after combined treatment (Figure 2). Liu et al. (2008) reported that the level of methamidophos resistance in *P. xylostella* declined sharply when exposed to high temperatures [40]. Previous studies have shown that temperature tolerance varies significantly between the different invasive populations, and the MED had higher tolerance to high temperatures than the MEAM1 [20]. Collectively, these results may provide some references for the competition and displacement between the two cryptic species. 

## 5. Conclusions

In summary, our results show that the expression levels of the two *BtCYP450s* could be up-regulated slightly with insecticide or high temperature, which was verified through RNAi experiments. However, under the combined stress of temperature and insecticide, *B. tabaci* MEAM1 showed a different response, and tolerance displayed no significant difference or even decreased under combined treatments in *B. tabaci* MEAM1. These results may provide a reference for exploring the competition between temperature-tolerance-mediated cryptic species and offer guidance for the safer and more efficient control of *B. tabaci* when using pesticides in the field, which will ultimately reduce the irrational use of insecticides.

## Figures and Tables

**Table 1:** Toxicity of abamectin (AB) and thiamethoxam (TH) in newly emerged *B. tabaci* adults at 24 h after exposure.

Insecticides	LC_50_/mg·L^−1^	95% Confidence Interval	Linear Fitting Equation	R^2^	Chi-Square	*p* Value
AB	0.1507	0.0572−0.2443	y = 2.4169x + 6.724	0.962	0.692	0.241
TH	2.1343	0.8256−3.4430	y = 2.5283x + 4.479	0.9355	1.732	0.213

## Data Availability

Data are contained within the article and Supplementary Material and will be made available on request.

## Supplementary Materials

The following supporting information can be downloaded at: https://www.mdpi.com/article/10.3390/insects15060399/s1, Table S1: Primers used in cloning and full-length cDNA amplification for *CYP450s*; Table S2: Characteristics of *CYP450s* in *B. tabaci*; Table S3: Inferred amino acid sequence of CYP450s in *B. tabaci* with its homologs from other insects.
