# Supplementary material for: Effect of High Temperature on Abamectin and Thiamethoxam Tolerance in Bemisia tabaci MEAM1 (Hemiptera: Aleyrodidae)
Source: Insects. 2024 May 29;15(6):399. doi: 10.3390/insects15060399 (PMC11203426; doi:10.3390/insects15060399)
Supplement: Supplementary file 1 [file insects-15-00399-s001.zip › Supplementary meterials.pdf]

Supplementary meterials

Table S1: Primers used in cloning and full-length cDNA amplification for *CYP450s*

Gene		Primer sequences (5'→3')	Annealing Temperature (°C)	Product (bp)
Primers for cDNA cloning				
BtCYP6k1	Forward	GGTGGACGAAAATTCTTGGG	59.4	694
	Reverse	CTCTTTGAAATAGCGCGGG	58.7	
	5'RACE primer	CGAAACTGTGAGGAAGAATGC	53.9	258
	3'RACE primer	TAATGGTGTTATGCGGAAGC	52.6	520
BtCYP305a1	Forward	GATCAGGTTCTTTGCCCCC	59	542
	Reverse	CCCATCGCTTCTTGTCGTT	58.3	
	5'RACE primer	TGGTGAAGGTGTTTGAGTGC	55.6	407
	3'RACE primer	GACCAGAAAGATTTTTAGACAGCG	53.7	278
Primers for qRT-PCR				
BtCYP6k1	Forward	TCTCTTCGTGGCAAAACCG	55.7	122
	Reverse	CTTTGACACCAAGCCCTGC	57.4	
BtCYP305a1	Forward	GTTTGGATGATGCGGTCGTG	57.2	96
	Reverse	TACGCATGGATTCGAGGAGC	57.5	
RPL29	Forward	TCGGAAAATTACCGTGAG	60	144
	Reverse	GAACTTGTGATCTACTCCTCTCGTG	60	
EF-1α	Forward	TAGCCTTGTGCCAATTTCCG	60	110

	Reverse	CCTTCAGCATTACCGTCC	60	
Primers for dsRNA synthesis				
dsBtCYP6k1	Forward	TAATACGACTCACTATAGGG TTTTGTCACCCCCAGTGCT	66.1	415
	Reverse	TAATACGACTCACTATAGGG AACGAGGCAGCCAAGGAGT	67.2	
dsBtCYP305a1	Forward	TAATACGACTCACTATAGGG AAAGGGAGAACATCTGCGTCAC	65.4	417
	Reverse	TAATACGACTCACTATAGGG GCGAGCCAGCAATGAAGAAA	66	
dsGFP	Forward	TAATACGACTCACTATAGGG AAGTTCAGCGTGTCCG	68.3	414
	Reverse	TAATACGACTCACTATAGGG CACCTTGATGCCGTTC	68.1	

Table S2: Characteristics of *CYP450s* in *B. tabaci.*

Cryptic species	Gene	cDNA size (bp)	ORF size (bp)	Molecular weight (kDa)	Isoelectric point	GenBank accession no.
MEAM1	BtCYP6k1	2401	1575	60.28	8.74	PP239342
	BtCYP305a1	2679	1503	56.58	8.87	PP239341

Table S3: Inferred amino acid sequence of CYP450s in *B. tabaci* with its homologs from other insects.

Species		Type	GenBank accession number
Hemiptera	Bemisia tabaci	CYP6k1	PP239342
	Bemisia tabaci	CYP305a1	PP239341

	Bemisia tabaci	CYP6a8	XP_018916753.1
	Bemisia tabaci	CYP6B7	XP_018916758.1
	Bemisia tabaci	CYP6B4	XP_018899928.1
	Bemisia tabaci	CYP6d3	XP_018916092.1
	Aphis gossypii	CYP305a1	XP_027843062.2
	Diuraphis noxia	CYP305a1	XP_015375411.1
	Myzus persicae	CYP305a1	XP_022183031.1
	Rhopalosiphum maidis	CYP305a1	XP_026808649.1
	Rhopalosiphum padi	CYP305A1	UOW66142.1
	Melanaphis sacchari	CYP305a1	XP_025194392.1
	Aphis craccivora	CYP305a1	QQL12328.1
Lepidoptera	Amyelois transitella	CYP6B2	XP_013186352.1
	Trichoplusia ni	CYP6B6	XP_026741446.1
Orthoptera	Schistocerca americana	CYP6a2	XP_047004894.1
	Schistocerca piceifrons	CYP6a2	XP_047096694.1
Thysanoptera	Thrips palmi	CYP6a14	XP_034236058.1
	Frankliniella occidentalis	CYP6a14	XP_026283616.1
Diptera	Phlebotomus papatasi	CYP6a9	XP_055697205.1
	Lutzomyia longipalpis	CYP6a9	XP_055696257.1
	Bradysia coprophila	CYP6d3	XP_037026731.1
	Sitodiplosis mosellana	CYP6d5	XP_055305823.1
	Uranotaenia lowii	CYP6d5	XP_055610461.1
	Phlebotomus papatasi	CYP305a1	XP_055701115.1
	Hermetia illucens	CYP305a1	XP_037908019.1
	Uranotaenia lowii	CYP305a1	XP_055609284.1
	Anopheles coustani	CYP305a1	XP_058122927.1
Coleoptera	Anthonomus grandis grandis	CYP6A1	XP_050305705.1
	Aethina tumida	CYP305a1	XP_019871644.2

Tenebrio molitor	CYP305A1	AKZ17688.1
Mylabris cichorii	CYP305a1	AMR44690.1
Tribolium castaneum	CYP305a1	XP_970235.1
Diabrotica virgifera virgifera	CYP305a1	XP_050508447.1
Diorhabda carinulata	CYP305a1	XP_057671993.1
Diorhabda sublineata	CYP305a1	XP_056630734.1
